# Supplementary material for: Specific intracellular signature of SARS-CoV-2 infection using confocal Raman microscopy
Source: Commun Chem. 2022 Jul 25;5:85. doi: 10.1038/s42004-022-00702-7 (PMC9311350; doi:10.1038/s42004-022-00702-7)
Supplement: Supplementary file 2 — Supplementary Information [file 42004_2022_702_MOESM2_ESM.docx]

**Supplementary data**

**Table SI: Significant Raman peaks observed in the PC loadings contributing to the separation of SARS-CoV-2 or MeV-infected and non-infected cells.**

| ***Raman peaks in cm^-1^** | | |
| --- | --- | --- |
| **Cytoplasm region** | **Golgi-mitochondria** | **Nucleus** |
| 465: glycogen | 790: phosphodiester bands in DNA O-P-O stretching DNA | 1006 : Phenylalanine |
| 782 : DNA, Thymine, cytosine, uracil, RNA, U, T, C (ring breathing modes in the DNA/RNA bases) | 1099: n(C-N) | 1240: RNA |
| 1001: Phenylalanine | 1253: A, T (ring breathing modes of the DNA/RNA bases ,lipid | 1304 : adenine, cytosine |
| 1442: Cholesterol, fatty acid band d(CH2) (lipids) | 1294: CH2 deformation (lipid) | 1457 :Deoxyribose d(CH2) |
| 1457: Deoxyribose d(CH2) | 1304: CH2 deformation lipid , | 1572: Ring breathing modes in the DNA bases G, A (ring breathing modes of the DNA/RNA bases) |
| 1651 Lipid (C=C stretch) | 1457 :Deoxyribose d(CH2) | 1651: (C=C) Amide I Protein |
| 2885: v3 CH3, lipids, fatty acids | 1584: C=C bending mode of phenylalanine | 2872 :CH_2_ asymmetric stretch and CH stretch of lipids and proteins |
| 2929: CH_2_ asymmetric stretch | 1646: Amide I | 2881 :CH2 asymmetric stretch of proteins |
|  | 1656: Amide I | 2927 :Symmetric CH3 stretch Due primarily to protein |
|  | 2849 :CH_2_  asymmetric stretch of lipids and proteins | 2941: C-H vibrations in proteins |
|  | 2862: CH_2_ symmetric stretch of lipids |  |
|  | 2906: C-H stretch of lipids and proteins |  |
|  | 2949: lipids, fatty acids |  |

***** Classification of the infection signature (either SARS-CoV-2 or MeV) compared to non-infected cells.


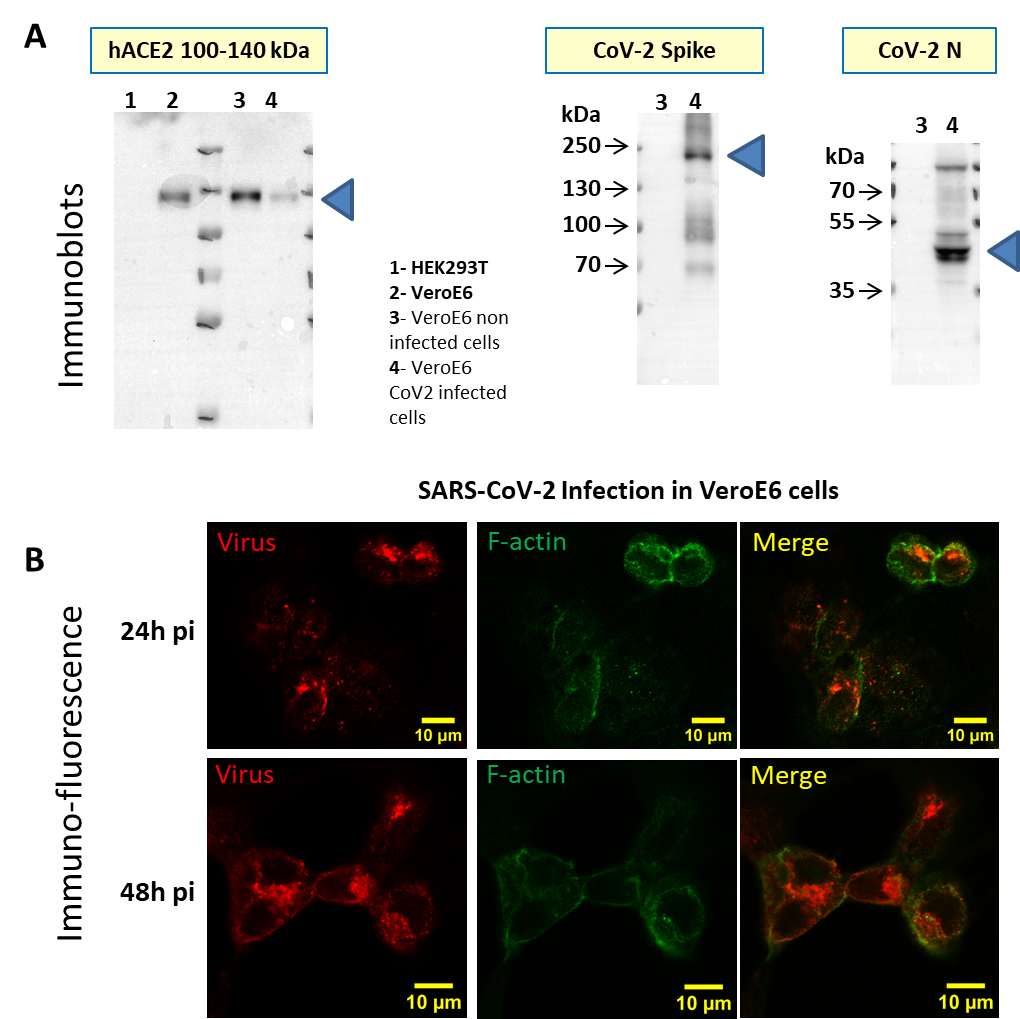


**
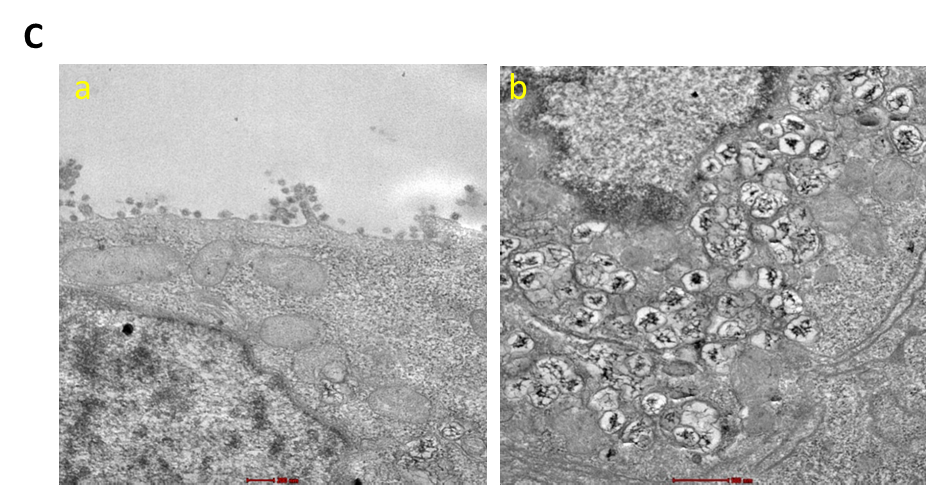
**

**B**

**Figure S1: Characterization of SARS-CoV-2 infected VeroE6 cells.**

1. Cellular human ACE2 and viral gene expression analysis of SARS-CoV-2 infected VeroE6 cells using immunoblots. Western-blot analysis for human ACE2 expression in HEK293T (lane 1) and in VeroE6 (lane 2) cell lysates, and in infected (lane 4) versus non-infected (lane 3) VeroE6 cells. Western blots of the SARS-CoV-2 Spike and Nucleocapsid N proteins in control (lane 3) and infected (lane 4) VeroE6 cells 48h post-infection with SARS-CoV-2 (Wuhan) at MOI= 0.1.
2. Immuno-fluorescence images of infected VeroE6 cells 24h or 48h post-infection with SARS-CoV-2 (Wuhan) using confocal fluorescence microscopy. For imaging the virus, rabbit anti-M primary antibody and then secondary antibody anti-rabbit Alexa568 (red) were used. For imaging the cells, F-actin labelling with Phalloidin-Alexa 488 (green) was used on fixed cells. The merged image is shown revealing all infected cells, in conditions similar to the confocal Raman spectroscopy experiment.
3. Transmission electron microscopy images of thin sections of SARS-CoV-2 infected VeroE6 cells producing SARS-CoV-2 showing (a) SARS-CoV-2 particles at the cell surface and (b) multiple intracellular replicative virus-filled organelles inside the cell cytoplasm that are absent in non-infected cells. Scale bars are 200-500 nm as indicated.


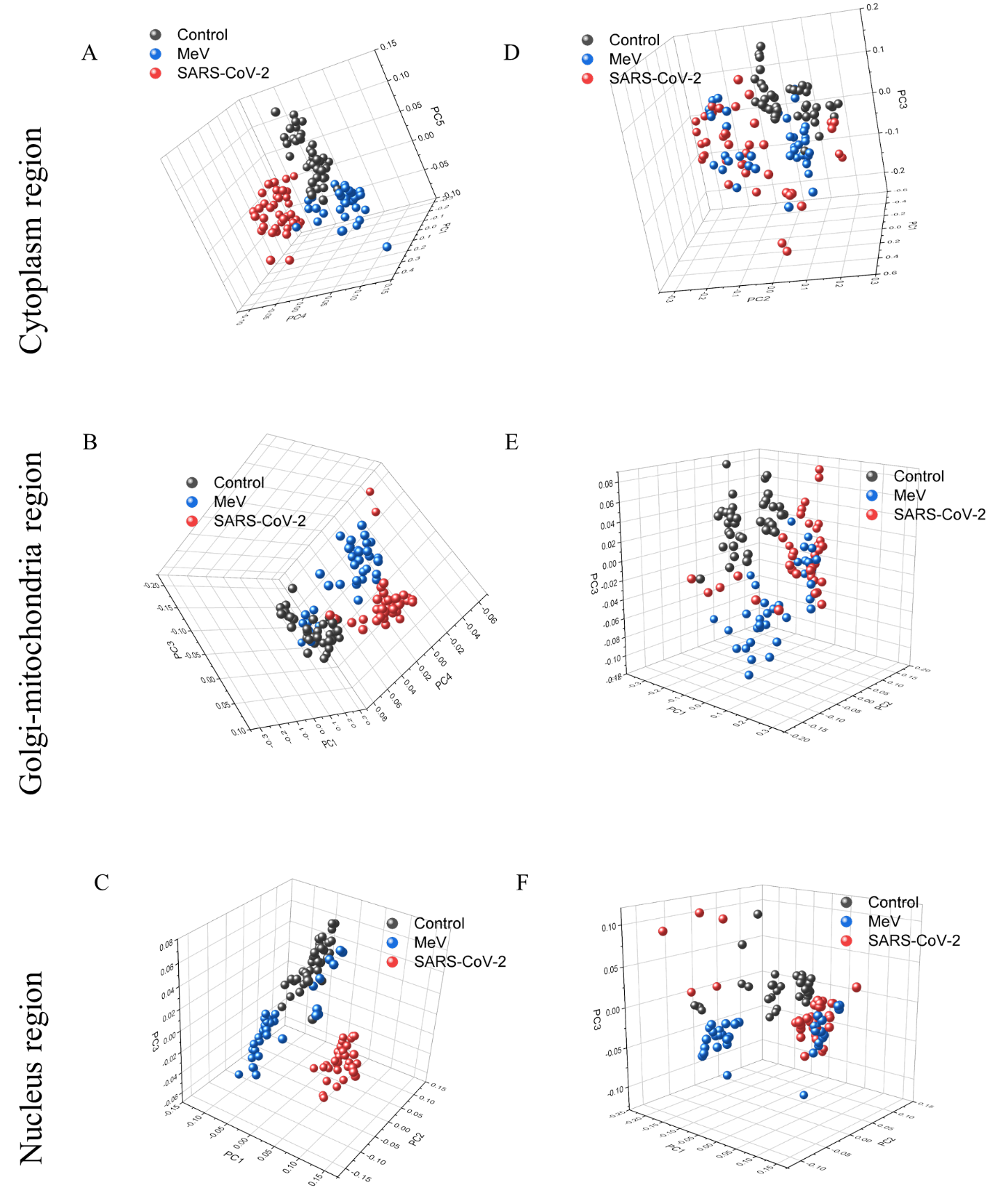


**Figure S2:** The score plot obtained using Principal component analysis for experimental batch 2 (A to C) and experimental batch 3 (D to F).

**
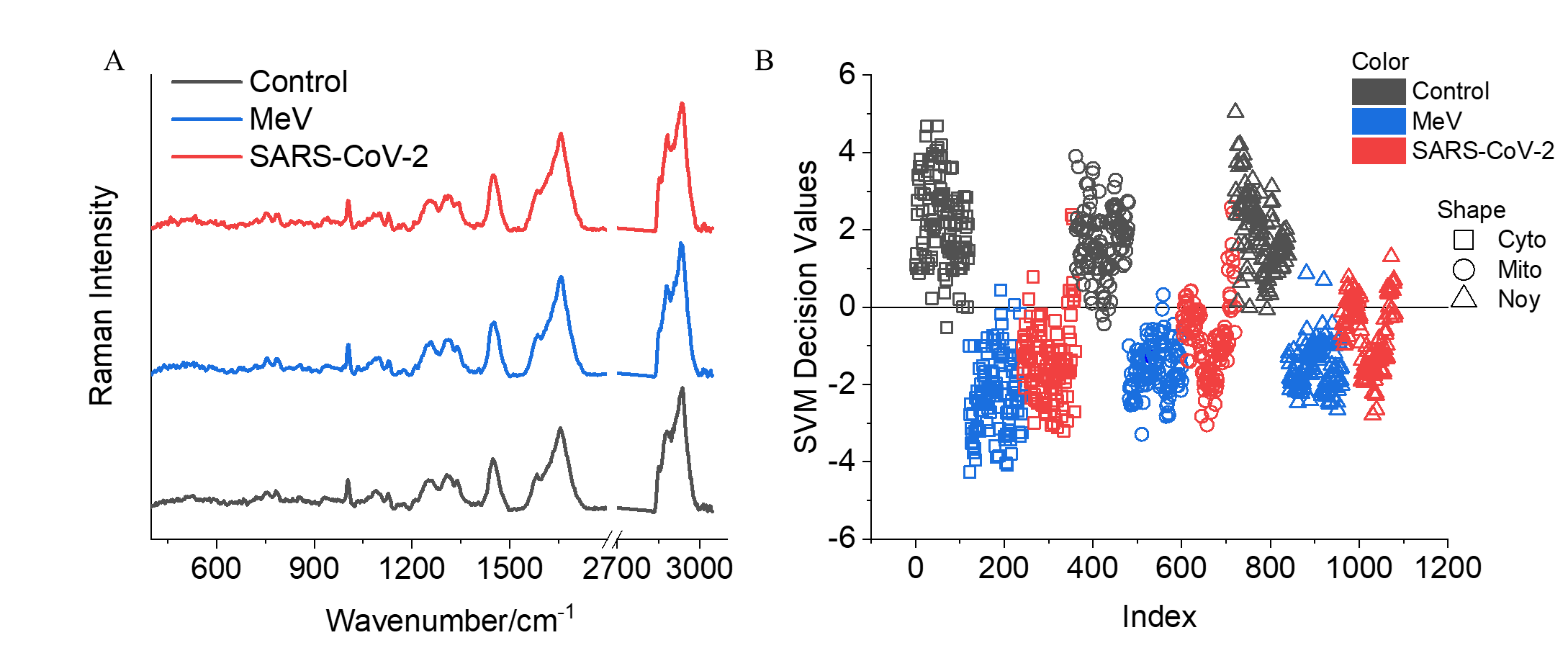
**

**Figure S3**: Raman model generated using support vector machine (SVM) algorithm to differentiate non-infected (Control, black) from SARS-CoV-2 (red) and measles virus (MeV, blue) infected Vero E6 cells. The model was generated using Raman spectra extracted from intracellular components: cytoplasm (Cyto, square), Golgi-mitochondria bodies (Mito, circle), and nucleus (Noy, triangle) of the Vero E6 cells. (Total classification accuracy 95%, 10-fold cross-validation)
